# Alleviation of symptoms and paradoxical brain perfusion shift in oral cenesthopathy: A retrospective neuroimaging study

**DOI:** 10.3389/fpsyt.2025.1653444

**Published:** 2025-09-02

**Authors:** Yojiro Umezaki, Haruhiko Motomura, Shigeki Nagamachi, Akira Toyofuku, Trang Thi Huyen Tu, Toru Naito

**Affiliations:** ^1^ Section of Geriatric Dentistry, Department of General Dentistry, Fukuoka Dental College, Fukuoka, Japan; ^2^ Japan Imaging Center of Psychiatry and Neurology, Fukuoka, Japan; ^3^ Department of Radiology, Faculty of Medicine, Fukuoka University, Fukuoka, Japan; ^4^ Department of Psychosomatic Dentistry, Graduate School of Medical and Dental Sciences, Institute of Science Tokyo, Tokyo, Japan; ^5^ Department of Basic Dental Sciences, Faculty of Odonto-Stomatology, University of Medicine and Pharmacy at Ho Chi Minh City, Ho Chi Minh, Vietnam

**Keywords:** oral cenesthopathy, oral dysesthesia, oral somatic delusions, cerebral blood flow, single photon emission computed tomography, coping behavior

## Abstract

**Introduction:**

Oral cenesthopathy is characterized by abnormal, distressing oral sensations without identifiable physiological or pathological causes. Right side predominant regional cerebral blood flow (rCBF) asymmetry in the broad brain area is suggested as pathophysiology of oral cenesthopathy. Coping behaviors, which provide temporary symptom relief, are sometimes employed by patients, but their effect on brain function is unclear. This study aims to examine changes in rCBF associated with coping behaviors.

**Methods:**

Seven patients with oral cenesthopathy were included in this study. Each patient underwent two single photon emission computed tomography (SPECT) scans: one during the state of discomfort (at steady state) and another after performing coping behaviors. SPECT data were analyzed using three-dimensional stereotactic regions of interest template, and Fine stereotactic regions of interest template.

**Results:**

At the steady state, right-side rCBF predominated in several brain regions, including the temporal lobe, without significant difference. During coping behaviors, this right-sided rCBF asymmetry was amplified, and showed significant differences, including the fusiform, lingual and parahippocampal gyri. These regions, associated with visual processing, suggest that oral cenesthopathy may involve more than just somatosensory dysfunction.

**Conclusion:**

Coping behaviors in oral cenesthopathy were associated with amplified right-sided rCBF asymmetry. This finding challenges the expectation that symptom alleviation would reduce rCBF asymmetry. It may suggest that the coping behavior of the patients with oral cenesthopathy is potentially amplifying asymmetry especially in the higher visual processing to adapt to their symptoms.

## Introduction

Cenesthopathy is a clinical entity first proposed by Dupré and Camus in the early 20th century (1) as “alterations of the common or internal sensibility, that is to say the disorders of these sensations which incessantly arrive at the brain from all the points of the body and which, in the normal state, do not impose themselves on our attention by any particular character, either in their intensity, or in their modality”. Patients with cenesthopathy commonly report vague, distressing, and often bizarre sensations, such as feelings of tightness, heat, or crawling under the skin, lacking an identifiable physiological or pathological basis. It often occurs in the oral region, where it is specifically referred to as oral cenesthopathy (2, 3). In cases of oral cenesthopathy, patients complain of discomfort such as stickiness or slurping in the mouth and abnormal sensations such as frog eggs or screws hanging out in the mouth. These symptoms can significantly impair quality of life, leading to functional disability and emotional distress (4).

Cenesthopathy has since been recognized as a complex phenomenon frequently associated with psychiatric conditions such as schizophrenia, depression, and anxiety disorders. On the other hand, it often occurs monosymptomatically, without being associated with other psychiatric disorders. This disorder of cenesthesia is an independent concept, distinct from hallucinations and delusions, and is a disturbance in the more primitive foundations of personality (1).

Despite its clinical relevance, the underlying mechanisms of oral cenesthopathy remain poorly understood. Recent advances in neuroscience suggest that oral cenesthopathy may be linked to abnormalities in central nervous system function (5–7). We have shown that the patients with oral cenesthopathy exhibit right-sided predominant regional cerebral blood flow (rCBF) asymmetries in broad area of the brain (8). We also reported that this left-right rCBF asymmetry was consistent with the presence or absence of a history of depression (9). Furthermore, we reported that this asymmetry was obscured in patients who improved with psychopharmacological treatment (10) and electro-convulsive therapy (11), emphasizing that right > left asymmetry of rCBF is associated with oral cenesthopathy.

From the perspective of content of complaints, the patients of oral cenesthopathy have a variety of complaints of discomfort and foreign body sensation as described above. At the same time, there is diversity, with some cases showing diurnal variability and regional mobility, and others showing improvement through coping behavior such as chewing gum or licking candy. For its management, it is likely that patients with oral cenesthopathy are often instructed to engage in these coping behaviors as an *ad hoc* solution, but the effect for brain function of this approach is unclear.

The objective of this study is to identify the change in rCBF associated with coping behavior through which patients with oral cenesthopathy experience temporary symptom improvement. Additionally, based on the findings, we aim to provide a potential mechanism of these coping behaviors.

## Methods

### Participants

The subjects were the patients with oral cenesthopathy in the outpatients of the Department of Geriatric Dentistry, Fukuoka dental college, Fukuoka, Japan, from April 2017 to March 2023. The diagnosis of oral cenesthopathy was made by a clinician certified by the Japanese Society of Psychosomatic Dentistry, based on the criteria proposed in the position paper regarding oral cenesthopathy and oral dysesthesia (12) after medical interviews and intra or extraoral examination. The data of patients with oral cenesthopathy, those who could improve their oral symptoms with coping behavior and requested SPECT was collected in this study.

The patients were excluded if cognitive deficits were detected, and the measured revised Hasegawa dementia scale (HDS-R) scores of the patients were <25. Although a cutoff of <21 is commonly used to screen for dementia (13), we adopted a stricter threshold to exclude patients with even mild cognitive impairment, to minimize heterogeneity in cerebral perfusion patterns. The patients with abnormalities on magnetic resonance image (MRI) of the brain were also excluded. This study was approved by the Ethics Committee of Fukuoka Dental College (approval no. 503).

### Brain perfusion single photon emission computed tomography

SPECT scans were scheduled within a month after the initial visit to our clinic and 2 weeks after the first scan. At the first SPECT scan, the subjects were requested to remain in a comfortable supine position with their eyes closed in a quiet room. All patients were in a steady state, meaning under continuous uncomfortable oral symptoms. At the second SPECT scan, all patients were at temporary alleviation of uncomfortable oral symptoms by each coping behavior during injection of 99mTc-ECD and monitoring passage. After confirming that the 99mTc-ECD was stabilized, the coping behavior was discontinued, and the patient was placed in a resting state during data acquisition. On both scanning periods, we confirmed that the complaints regarding oral cenesthopathy itself remained unchanged from the initial visit, despite temporary improvement by coping behavior.

The data acquisition process was initiated following the administration of a 600 MBq bolus injection of 99mTc-ECD via the right brachial vein. Initially, the passage from the heart to the brain was monitored using a rectangular large-field, dual-head gamma camera (E.CAM Signature; Toshiba, Tokyo, Japan) equipped with low-energy, high-resolution, parallel-hole collimators. The data acquisition process entailed the capture of a sequence comprising 100 frames, with a temporal resolution of 1 second per frame, using a 128 × 128 matrix. Subsequently, five minutes after the injection of 99mTc-ECD, SPECT images were obtained utilizing the aforementioned gamma camera, which was equipped with fan-beam collimators. The energy window was set at 140 keV ± 15%, and 45 step-and-shoot images were obtained throughout 180 degrees of rotation with an acquisition time of 30 s/step. All images were reconstructed using the ordered subsets expectation maximization (OSEM) method and subsequently smoothed three-dimensionally using a Butterworth filter. The reconstructed images were corrected for gamma ray attenuation using the Chang method.

### Statistical analysis

All data, including sex, age, duration of illness, and results of SPECT scan, were collected retrospectively. The rCBF quantification was performed using three-dimensional stereotaxic regions of interest (ROI) template (3DSRT) and fine stereotaxic ROI template (FineSRT), in which rCBF values were presented with subdivided values from 24 segments (12 segments in each hemisphere) in 3DSRT into 92 segments (46 segments in each). 3DSRT and FineSRT are fully automated rCBF quantification programs that can be used for examining a total of 636 ROIs. These 636 ROIs are categorized into 12 brain segments on the 3DSRT template: callosomarginal, precentral, central, parietal, angular, temporal, posterior cerebral, pericallosal, lenticular nucleus, thalamus, hippocampal, and cerebellar segments. In the same way, the 636 ROIs are categorized into 46 brain segments on the FineSRT template: superior frontal, medial frontal, paracentral lobule, anterior cingulate, subcallosal, orbital, rectal, middle frontal, inferior frontal, precentral, postcentral, insula, superior parietal, inferior parietal, supramarginal, angular, superior temporal, middle temporal, inferior temporal, transverse temporal, superior occipital, middle occipital, inferior occipital, precuneus lower, cuneus, hippocampus, fusiform, lingual, parahippocampal, amygdaloid body, thalamus, putamen, globus pallidus, caudate head, caudate tail, precuneus upper, cingulate, posterior cingulate, vermis, anterior lobe, posterior lobe, hypothalamus, quadrigeminy, substantia nigra, nucleus ruber and pons. The blood flow to each ROI was quantified in mL/100 g/min.

For the quantitative analysis, the rCBF values obtained from the 3DSRT and FineSRT data were used. In order to observe the balance/imbalance of left to right rCBFs, paired T-tests were performed at steady state and during coping behavior, respectively. All of the analysis of 3DSRT and FineSRT were performed using SPSS version 29.0.1.0 (IBM, Armonk, NY).

## Results

### Patients’ demographics and clinical measures

Data from seven patients with oral cenesthopathy were collected. **Table 1** presents the demographic characteristics, the type of coping behavior employed, and the medication status at both baseline and during the coping behavior scan. The patients consisted of a man and 6 women ranging in age from 35 to 77 (mean; 61.14, SD; 13.81) years old and in duration of illness from 3 to 48 (mean; 15.57, SD; 18.41) months. The patients’ complaints were limited to the oral area, with no hallucinations or delusions in other body parts. Although 4 patients had histories of psychiatric disorder, their psychiatric conditions were under remission. All of the patients were socially independent, and no cognitive decline was detected. All patients were right-handed, which was confirmed using Edinburgh inventory (14). Coping behaviors were variable including drinking water, denture wearing, gum chewing and candy licking. In common, the patients felt more than 50% improvement in VAS during the coping behaviors.

**Table 1:** Demographic and clinical characteristics, including coping behaviors, of patients with oral cenesthopathy.

Case no.	Age	Sex	Occupation	History of psychiatric disorders	Duration of illness (months)	Symptoms of initial visit	Coping behavior	Medication (/day)
1	61	Female	Part-time worker	Bipolar disorder	6	Upper and lower lip mucosa are sticky	Drinking water	None
						Puffy feeling		
2	35	Female	Nurse	Depression	36	Sticky stuff stuck in the mouth	Wearing mouthpiece	Aripiprazole (1 mg)
3	70	Female	Factory worker	Depression	48	Air comes out of the oral mucosa	Gum chewing	Mirtazapine (15 mg)
						Sticky saliva		
						Burning sensation in the buccal mucosa		
4	77	Female	Housewife	None	6	Slimy sensation in the mouth	Wearing denture	None
5	63	Female	Office worker	None	3	Sensation of blisters in the mouth	Drinking water	None
6	51	Male	Office worker	Depressive state	5	Sticky sensation in the mouth	Gum chewing	None
7	69	Female	Employee of non-gevernmental organization	None	5	Sticky or coarse feeling in the mouth	Candy licking	Aripiprazole (1 mg)

### The changes of balance of left to right rCBF by coping behaviors

**Figure 1:**
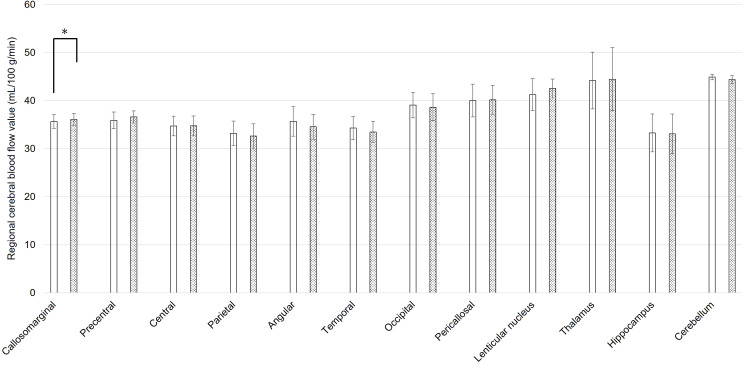
Regional cerebral blood flow values for each brain area using three-dimensional stereotaxic regions of interest (ROI) template, at the static state of oral cenesthopathy. The X-axis shows each region, and the Y-axis shows rCBF values. The graphs on the left (white bars) and right (doted bars) show the mean and SD for the right and left rCBF value, respectively. The ROIs with a significant difference of the ratio are denoted by a single asterisk (P<0.05).

**Figure 2:**
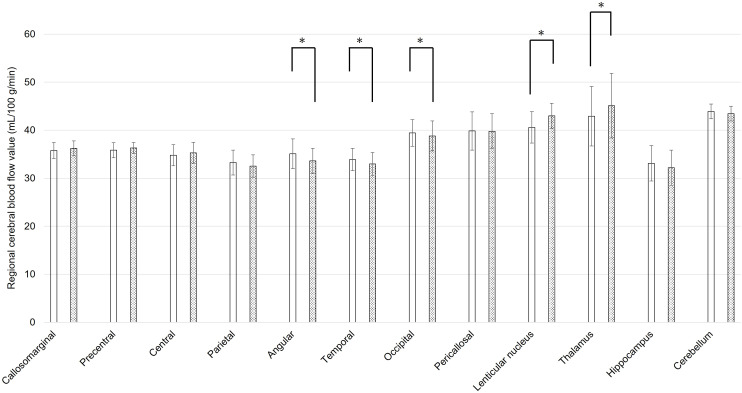
Regional cerebral blood flow values for each brain area using three-dimensional stereotaxic regions of interest (ROI) template, during coping behavior of oral cenesthopathy. The X-axis shows each region, and the Y-axis shows rCBF values. The graphs on the left (white bars) and right (doted bars) show the mean and SD for the right and left rCBF value, respectively. The ROIs with a significant difference of the ratio are denoted by a single asterisk (P<0.05).

The mean rCBF values of static state and during coping behavior in 3DSRT were shown in **Figures 1**, **2**, respectively. At the static state, the significant difference between right and left rCBF was detected only in callosomarginal area, with left-side predominance. Right-side predominant rCBFs were detected in parietal, angular, temporal, occipital, hippocampus and cerebellum area, without statistically significant differences. On the other hand, at the coping behavior, angular, temporal lobe, occipital, lenticular nucleus and thalamus had significant differences between right and left rCBFs. In detail, lenticular nucleus and thalamus areas showed left-side predominant, and angular, temporal lobe and occipital areas showed right-side predominant rCBFs.

**Tables 2**, **3** show the cerebral regions with significant right-left differences in rCBF values using FineSRT, at static state and during coping behavior, respectively. At the static state, significant left-side predominant rCBFs were detected in superior frontal area. Significant right-side predominant rCBFs were detected in angular and posterior cingulate areas (in total 2 areas). During coping behavior, medial frontal, thalamus, globus pallidus and vermis areas showed significantly left-side predominant rCBFs. Significant right-side predominant rCBFs were detected in orbital, angular, superior temporal, middle temporal, inferior occipital, fusiform, lingual, parahippocampal and posterior cingulate areas (in total 9 areas).

**Table 2:** Regional cerebral blood flow values of the patients with oral cenesthopathy in static state.

Cerebral regions	Right	Left	*P*
SuperiorFrontal	33.17 ± 1.24	< 33.92 ± 1.12	0.028*
Angular	35.83 ± 3.08	> 34.53 ± 2.59	0.043*
PosteriorCingulate	43.97 ± 5.51	> 41.75 ± 4.78	0.018*

*Statistically significant difference in rCBF values between the right and left brain regions (p < 0.05).

**Table 3:** Regional cerebral blood flow values of the patients with oral cenesthopathy during coping behavior.

Cerebral regions	Right	Left	*P*
MedialFrontal	38.01 ± 1.28	< 38.82 ± 1.05	0.028*
Orbital	35.78 ± 2.96	> 34.12 ± 3.35	0.018*
Angular	35.24 ± 3.11	> 33.60 ± 2.60	0.018*
SuperiorTemporal	33.68 ± 3.26	> 32.79 ± 3.18	0.043*
MiddleTemporal	34.05 ± 2.09	> 32.81 ± 2.27	0.028*
InferiorOccipital	34.92 ± 2.11	> 33.51 ± 1.48	0.043*
Fusiform	40.23 ± 1.72	> 39.15 ± 1.43	0.028*
Lingual	44.36 ± 3.29	> 42.79 ± 3.66	0.028*
Parahippocampal	36.32 ± 3.23	> 34.54 ± 2.95	0.018*
Thalamus	43.03 ± 6.20	< 45.19 ± 6.68	0.043*
GlobusPallidus	34.98 ± 2.12	< 38.44 ± 1.73	0.018*
PosteriorCingulate	44.48 ± 7.71	> 41.22 ± 6.71	0.018*
Vermis	44.48 ± 1.75	< 45.51 ± 2.17	0.018*

*Statistically significant difference in rCBF values between the right and left brain regions (p < 0.05).

## Discussion

In this study, we analyzed changes in cerebral blood flow in patients with oral cenesthopathy due to coping behavior. In the 3DSRT analysis, right-sided predominance in rCBF was observed in the broad area including temporal lobe in the steady state, although the differences were not significant. During coping behavior, significant right-sided predominance in rCBFs were observed in the temporal lobe and other areas. Using FineSRT analysis, significant rightward rCBF asymmetries were observed in 2 areas (angular and posterior cingulate) in the steady state, with asymmetries observed in 9 areas (orbital, angular, superior temporal, middle temporal, inferior occipital, fusiform, lingual, parahippocampal and posterior cingulate) during the coping behavior. In common with the 3DSRT and FineSRT analyses, it was suggested that right-sided rCBF dominance in the broad areas including temporal lobe during the steady state may be amplified during coping behavior.

In this study, we employed both the 3DSRT and FineSRT ROI templates to analyze rCBF patterns. The 3DSRT template, which divides the brain into 12 bilateral segments, is suitable for observing general hemispheric trends and major anatomical regions. In contrast, the FineSRT template offers a more granular parcellation into 92 regions per hemisphere, allowing us to detect more localized functional asymmetries, particularly in areas related to higher-order processing such as the fusiform and parahippocampal gyri. While these templates are not entirely independent, we found that using both allowed us to characterize rCBF asymmetry at both macro- and micro-levels, enhancing the robustness of our findings. We acknowledge that overlapping findings across the two templates strengthen the interpretability of the results. We also explored hemispheric asymmetry using laterality indices (right/right+left ratios). Although statistically significant changes in laterality indices were observed in a few regions such as the anterior cingulate gyrus, rectal gyrus, and hypothalamus, these findings were not easily interpretable in the context of oral cenesthopathy or coping mechanisms. Therefore, we chose to focus on region-based analysis, emphasizing the number and nature of brain regions that exhibited significant left-right rCBF differences, which we believe provides a more clinically meaningful and neurobiologically relevant perspective.

The finding of a right-sided predominance rCBF in broad brain regions, including the temporal lobe, in patients with oral cenesthopathy in static state was consistent with our previous report (8). We originally hypothesized that rCBF asymmetry would be attenuated during temporal improvement of symptoms of oral cenesthopathy by coping behavior by analogy with previous cases in which psychopharmacotherapy (10) and electroconvulsive therapy (11) had been successful. However, the results of the present study indicate that the rCBF asymmetry is instead amplified during coping behavior, indicating that the opposite of original hypothesis is detected.

Among the various regions in which the rCBF asymmetry became more apparent due to coping behaviors, some regions have interesting features: temporal (superior temporal and middle temporal), fusiform, lingual, parahippocampal, and inferior occipital have a right-sided predominance. In particular, fusiform, lingual, parahippocampal, and inferior occipital are thought to be involved in higher-order processing of visual information. At first glance, the coping behaviors in this study, such as drinking water, wearing a mouthpiece, chewing gum, and licking candy, seem unrelated to vision. But why do these areas related to visual processing seem to be involved?

Focusing on the fusiform, this gyrus is a part of ventral temporal cortex, located between lingual gyrus and parahippocampal gyrus, and the inferior temporal gyrus. Although the all functions of fusiform remains unclear, it has many seemingly different proposed functions (15). Initially, the fusiform gyrus was reported to play an important role in face perception (16), but in recent years it has been reported to be associated with a variety of functions related to visual information, including categorical recognition, semantic understanding and combining multiple stimuli (17). Among them, the concept of “visual mental imaginary (VMI)” may be most worth considering in this study. VMI implicates representations and the accompanying experience of visual information without a direct external stimulus. Such representations, which are created by many processes such as retrieval, modifications, and recombination of sensory information from long-term memory, are recalled by various triggers. According to previous studies about VMI, the left fusiform gyrus plays an important role as a central hub for VMI (18). Patients with oral cenesthopathy complain about their distress symptoms repetitively and often clearly describe the inside of the mouth as if it is visible. For example, a patient said “A foreign material is wrapped around my teeth”, while bringing and showing us a photorealistic drawings of their mouths with troubling symptoms (19). These cases imply us that they have a different visual image about inside the mouth than reality. Repetitive consideration and perception of oral symptoms may lead to gradual solidification of a false visual image. Thus, the result in this study showing left<right perfusion imbalance especially in fusiform gyrus, may reflect that attenuation of the falsely acquired visual images, bringing a temporary relief of cenesthopathy symptoms.

In addition to the fusiform gyrus, several other regions showed significant right-sided rCBF predominance during coping behavior. The parahippocampal gyrus and lingual gyrus, both involved in higher-order visual and memory-related processing (18), may support the hypothesis that internal imagery or self-referential processing is engaged during coping. The angular gyrus showed significant right-sided rCBF predominance both in the steady state and during coping behavior with FineSRT. This suggests that its involvement may not be specific to coping, but rather a fundamental feature of the neural alterations underlying oral cenesthopathy itself. The angular gyrus plays a central role in multisensory integration, including visual, auditory, and tactile information, and contributes to body state perception and self-location (20). Its consistent right-dominant activation may reflect persistent alterations in the integration of oral somatosensory input and body perception, which are characteristic of this condition.

Considering the results, the asymmetry in rCBF in patients with oral cenesthopathy may not be simply representing the pathophysiology of the syndrome, but rather the result of patients’ engagement in coping with these unusual oral sensations -possibly by reinforcing internal representations or weakening false visual image that was caused by some other reason. In this context, coping behaviors may serve not to suppress but to modulate or even amplify existing functional asymmetries as an adaptive strategy. This interpretation highlights the complex neurofunctional mechanisms involved in symptom regulation, and underscores that increased asymmetry does not necessarily indicate worsening of the condition, but may reflect an active attempt to manage sensory abnormalities. Given the small sample size and exploratory design, we cannot determine whether the increased asymmetry reflects a maladaptive response or an adaptive coping mechanism. Nonetheless, both interpretations underscore the complex interplay between behavioral strategies and brain function in oral cenesthopathy. In other words, the rCBF asymmetries we have previously reported in oral cenesthopathy may represent a hybrid phenomenon: one component reflecting changes driven by mechanisms close to the primary cause of the cenesthopathy itself, and another arising secondarily as a neural adaptation to an abnormal internal state.

It is important to acknowledge the limitations of this study. Firstly, the number of cases included in the study is relatively small. Although brain perfusion SPECT was necessary to detect abnormalities on brain imaging and to assess the role of coping behavior in clinical practice for individuals, some patients declined to undergo the test due to concerns about its invasiveness of radioisotopes. Consequently, the SPM analysis did not detect significant results (data not shown). In addition, corrections for multiple comparisons were not applied due to the small sample size and exploratory nature of the study. Therefore, the present findings should be interpreted with caution. However, it is believed that important findings were obtained through the use of 3DSRT and FineSRT analysis. Secondly, this study was not concerned with oral cenesthopathy in its entirety. In this study, we are collecting cases in which “bizarre oral symptoms of temporary improvement can be achieved with coping behaviors”. There are many cases in which all coping behaviors are ineffective. Hence, different studies are needed to understand the pathophysiology of oral cenesthopathy itself.

In conclusion, the current study using 99mTc-ECD SPECT, analyzed by 3DSRT and FineSRT, revealed that right > left rCBF asymmetries in temporal lobe were amplified by coping behavior which produce temporary improvement in the patients with oral cenesthopathy. The observed change of rCBF in fusiform and other related areas imply the association of oral cenesthopathy and visual function. The rCBF asymmetry in oral cenesthopathy may reflect patients’ coping mechanisms, potentially amplifying asymmetry to adapt to their symptoms. Further study is needed to understand the pathophysiology of oral cenesthopathy in its entirety.

## Data Availability

The raw data supporting the conclusions of this article will be made available by the authors, without undue reservation.
